# Case Report: fNIRS-guided rehabilitation in refractory post-traumatic dysphagia

**DOI:** 10.3389/fresc.2025.1712962

**Published:** 2025-11-26

**Authors:** Xiaohan Wang, Liying Guo, Yanan Wang, Wenlei Ma, Lijin Wang, Yanning Yan, Yu Yin

**Affiliations:** 1 Department of Rehabilitation Medicine, Hebei Provincial People's Hospital, Shijiahzuang, China; 2 Hebei Provincial Key Laboratory of Cerebral Networks and Cognitive Disorders, Hebei General Hospital, Shijiazhuang, China

**Keywords:** severe traumatic brain injury, dysphagia, functional near-infrared spectroscopy (fNIRS), precision assessment, neurorehabilitation

## Abstract

**Background:**

Dysphagia is a common complication of severe traumatic brain injury (sTBI) and may persist despite standard treatment. For refractory cases, there appear to be no more effective therapeutic options available.

**Case information:**

A 15-year-old boy continued to suffer from severe dysphagia nine months after severe traumatic brain injury, remaining dependent on tracheostomy and nasogastric feeding. Prior interventions, including two botulinum toxin injections and a 35-day multimodal rehabilitation program, failed to yield meaningful improvement. At readmission, swallowing assessments showed profound impairment, malnutrition, and poor treatment tolerance. Functional near-infrared spectroscopy (fNIRS) was then applied to map cortical activation and guide targeted therapy. After 42 days, swallowing function improved significantly, with the Functional Oral Intake Scale (FOIS) rising from level I to VI and the Penetration-Aspiration Scale (PAS) decreasing from 8 to 2.

**Conclusions:**

The therapeutic outcome of this case suggests that selecting training methods based on fNIRS-identified cortical activation can have a positive and significant impact on swallowing rehabilitation in patients with severe traumatic brain injury.

## Introduction

Traumatic brain injury (TBI) is a leading cause of long-term neurological disability in both adults and adolescents, and post-TBI dysphagia remains one of its most debilitating sequelae, with prevalence estimates ranging from 27% to over 60% in hospitalized cases (1, 2). Dysphagia in the context of TBI differs significantly from stroke-related swallowing disorders in terms of underlying neurophysiology, lesion distribution, recovery trajectory, and therapeutic responsiveness (3).

The mechanisms contributing to post-TBI dysphagia are multifactorial, including direct cortical and subcortical injury, diffuse axonal damage, brainstem involvement, and secondary effects of tracheostomy, prolonged intubation, and ventilatory dependence (4). Clinical manifestations can include impaired oral containment, delayed swallow initiation, reduced pharyngeal contraction, and impaired airway protection, leading to malnutrition, dehydration, and recurrent aspiration pneumonia (5). In severe cases, patients remain dependent on tracheostomy and enteral feeding for extended periods, substantially impairing quality of life and rehabilitation potential.

While conventional rehabilitation approaches—such as sensory stimulation, neuromuscular electrical stimulation, and compensatory swallowing strategies—can benefit many patients, a subset of individuals exhibit refractory dysphagia despite intensive therapy (6). This population often requires precision-guided, neurophysiologically informed interventions. Functional near-infrared spectroscopy (fNIRS) offers a non-invasive means of monitoring cortical activation patterns in real time, enabling clinicians to individualize treatment strategies based on objective neural response profiles (7).

Here, we report a complex case of severe post-TBI dysphagia refractory to standard rehabilitation and pharmacologic management, in which fNIRS-guided therapy facilitated clinically meaningful recovery.

## Case presentation

A 15-year-old male sustained a severe traumatic brain injury in a motor vehicle accident on June 27, 2024. CT and MRI scans of the head revealed a localized contusion in the brainstem (midbrain and pons), accompanied by a right frontal-temporal subdural hematoma. Emergency surgical interventions included burr hole drainage for intracranial hematoma and tracheotomy. Nine months postoperatively (March 3, 2025), he was transferred to the Department of Rehabilitation at our hospital. Upon admission, the patient remained dependent on tracheostomy and nasogastric tube feeding. No history of infectious diseases, chronic diseases or family history. Unmarried and childless.

Neurological examination revealed that he was alert and fully conscious (Glasgow Coma Scale score: 15). Cranial nerve assessment showed symmetrical forehead wrinkling, a slightly flattened right nasolabial fold, and leftward deviation of the oral commissure. Involuntary jaw movements were observed in the mandibular region, characterized by rhythmic masseter contractions accompanied by audible teeth grinding. These movements diminished at rest but intensified with activity. No temporomandibular joint clicking was noted. Swallowing function was severely impaired.

Muscle strength was graded as 3/5 in the left upper limb and 0/5 in the right upper limb and both lower limbs. Bilateral pathological reflexes were absent.

**Figure 1:**
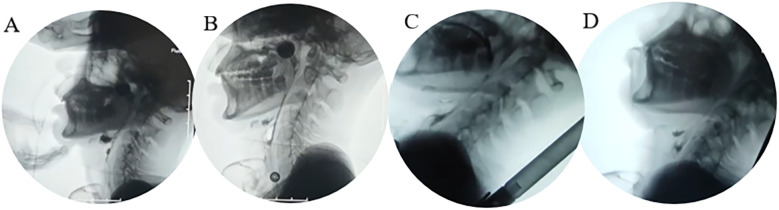
Four-panel video fluoroscopic swal. **(A)** Baseline (large food residue in the epiglottic groove); **(B)** D35 (most food residue cleared in the epiglottic groove); **(C)** D49 (safe feeding in semi-recumbent position); **(D)** Late pharyngeal phase (food not fully entering the esophagus, risk of aspiration).

**Figure 2:**
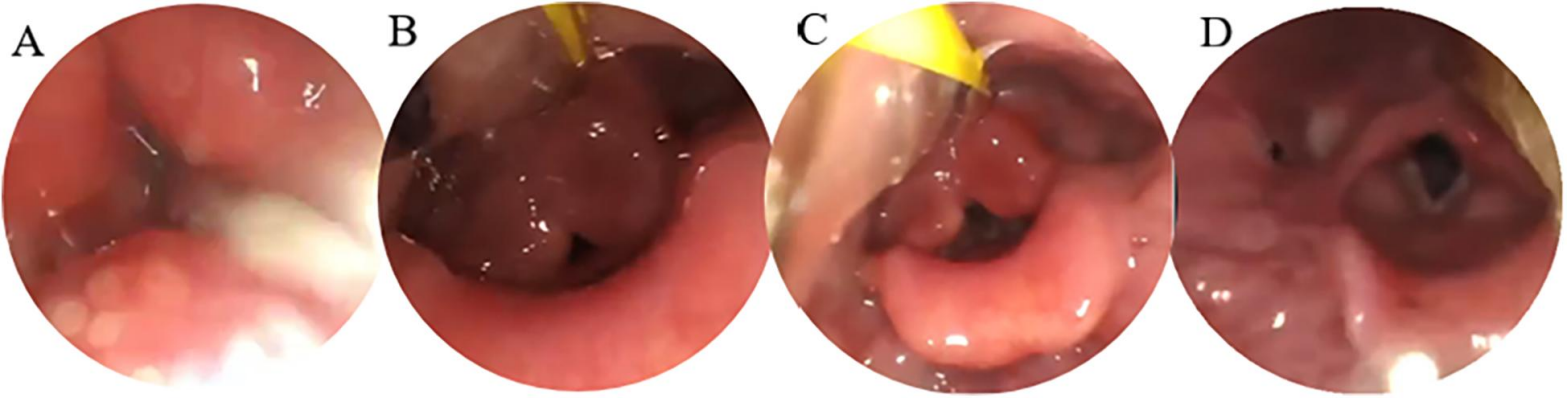
Four-view FEES examination. **(A)** Resting state (with abundant secretions in the pharynx); **(B)** pre-swallowing (with food bolus approaching the glottis); **(C)** swallowing (with incomplete glottis closure); **(D)** post-swallowing (with residual secretions in the pharynx).

Speech-Language Pathology Assessment. Dysarthria: The Frenchay Dysarthria Assessment–Second Edition (FDA-2) (8) indicated severely impaired function across multiple domains, with oral-facial structures, respiratory support, laryngeal function, and associated reflexes all rated at level E (Supplementary Table S1). Dysphagia: Clinical screening revealed a grade V on the Water Swallow Test (WST), and a level I on the Functional Oral Intake Scale (FOIS), indicating complete dependence on enteral feeding. Drooling severity was scored as 4, indicating profuse drooling. Unstimulated salivary flow rate was measured at 0.45 g/min (Supplementary Table S2). Instrumental Assessment: Video fluoroscopic Swallowing Study (VFSS) (Figure 1) confirmed severe cricopharyngeal dysfunction in the patient, characterized by food retention in the pharynx and subsequent aspiration. Modified Barium Swallow Impairment Profile (MBSImP) composite score was 49. Penetration-Aspiration Scale (PAS) score was 8. Quantitative analysis using ImageJ software showed: Inability to initiate oral bolus transport, Prolonged pharyngeal transit time of 120 s, Hyoid bone displacement at 110% of the C2–C4 distance, Pharyngeal constriction ratio of 34% (Supplementary Table S3). Fiberoptic Endoscopic Evaluation of Swallowing (FEES): Murray secretion severity scale: grade 3 (severe) (Figure 2), Fiberoptic Endoscopic Dysphagia Severity Scale (FEDSS): score of 6 (Supplementary Table S4).

## Intervention

Based on the patient's preliminary clinical evaluation, an individualized multimodal rehabilitation program was developed. This protocol included pharmacological treatment, botulinum toxin injections into the salivary glands, oropharyngeal sensory stimulation and motor training, neuromuscular electrical stimulation (NMES), and surface electromyography (sEMG) biofeedback training. The handheld low-frequency electrical stimulator (LGT-2350A, Guangzhou Longzhijie Medical Equipment Co., Ltd.) is operated by trained medical professionals. During treatment, electrodes are applied directly to target muscle groups including the lips, tongue, soft palate, and posterior pharyngeal wall. The treatment parameters are set as follows: voltage 6 V, frequency 5 Hz, pulse width 100 µs. The intensity should be adjusted to induce significant muscle contraction while ensuring patient tolerance, with sessions lasting 10 min and administered once daily.

The patient received two botulinum toxin injections (A-type) at days 7 and 21 post-admission, with a total dose of 100 U. The distribution was as follows: 30 U each to both parotid glands (2 injection sites per side in the middle of the superficial lobes) and 20 U each to both submandibular glands (1 injection site per side at the gland center). However, clinical observation showed no significant improvement in drooling symptoms following either injection.

Thirty-five days after the intervention, repeated measures were conducted to quantify rehabilitation outcomes. Results indicated no significant improvement in the Modified Barium Swallow Impairment Profile (MBSImP) total score. However, body mass index (BMI) declined significantly from 16.78 ± 0.5 to 16.15 ± 0.4 kg/m^2^ (*p* = 0.012). In addition, treatment tolerance time decreased from 87 ± 3 min to 43 ± 2 min (*p* < 0.01), suggesting progressive nutritional deterioration and a marked reduction in rehabilitation tolerance during the intervention period.

To further explore the patient's central nervous system compensatory mechanisms and optimize the individualized rehabilitation strategy, functional near-infrared spectroscopy (fNIRS) was employed (NirSmart system, Danyang Huichuang Medical Devices Co., Ltd., China). In this study, functional near-infrared spectroscopy (fNIRS) was employed as an assessment tool rather than a real-time feedback tool after 35 days of intervention. During the treatment process, fNIRS signals were not used to guide the patients. This technique enabled real-time monitoring of cortical activation during swallowing tasks, providing neurophysiological evidence to support precision rehabilitation planning.

**Figure 3:**
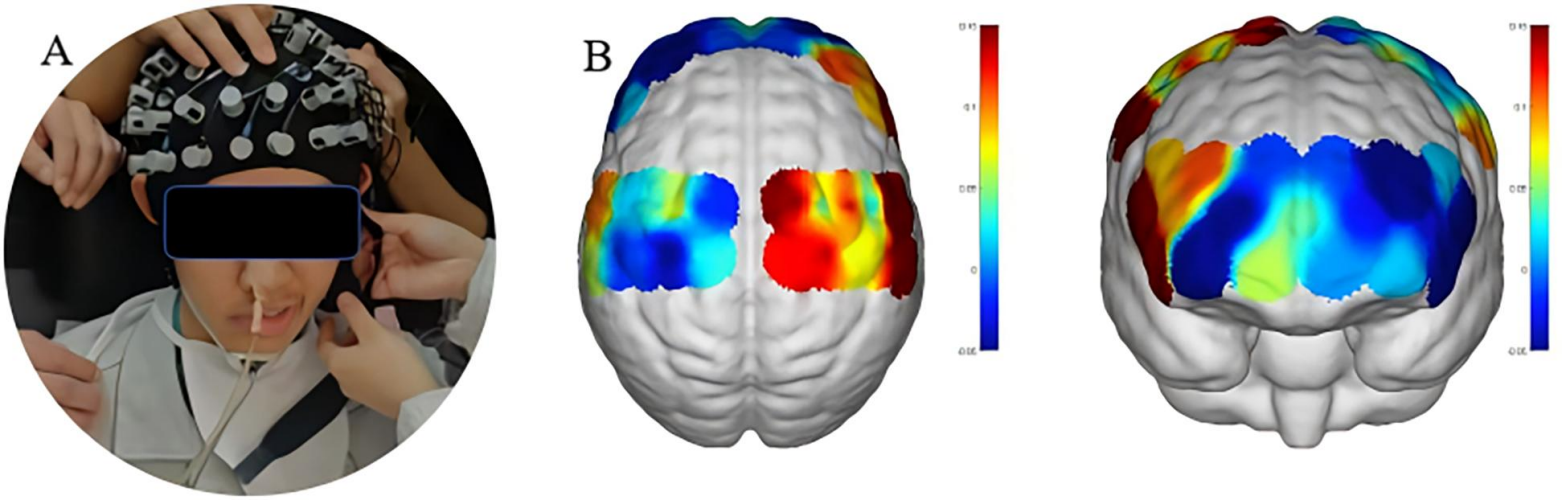
fNIRS setup and channel coverage. **(A)** Probe cap image with 19 emitters (red dots) and 13 detectors (blue dots). **(B)** Brain coronal view schematic showing coverage of 39 effective channels (bilateral frontal lobe BA4/6 and parietal lobe BA7/40).

**Figure 4:**
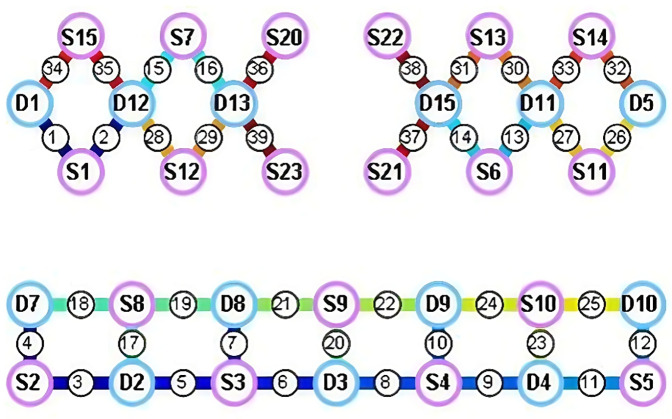
fNIRS cortical during activation. The purple circle represents the light source probe, which primarily emits light signals. The blue circle corresponds to the detection probe, responsible for measuring the intensity of the emitted light and converting it into electrical signals. The black circle indicates a measurement channel formed by the combination of a light source probe and a detection probe, with the corresponding brain region being the target area under study.

The fNIRS system used near-infrared light sources with wavelengths of 730 nm and 850 nm and a sampling rate of 11 Hz. The experimental setup consisted of 19 emitters and 13 detectors, forming 39 valid optical channels through an optimized probe layout (Figure 4). The monitoring area included bilateral prefrontal and parietal cortices, with spatial distribution of optodes shown in the corresponding Figure 3.

Data analysis was conducted using NirSpark software (Danyang Huichuang Medical Devices Co, Ltd, China). Motion artifacts were identified as signals exceeding six standard deviations from the mean and corrected using interpolation functions. A band-pass filter was applied to eliminate high-frequency physiological noise (>0.2 Hz) and low-frequency baseline drift (<0.01 Hz). Light intensity signals were converted to oxygenated hemoglobin (HbO2) concentrations using the modified Beer–Lambert law. In the second phase (D36-D77), fNIRS applications were divided into two stages: ① Baseline assessment (with a single cortical activation mapping session at D35): Patients completed swallowing-related tasks (e.g., active swallowing, swallowing triggered by sensory electrical stimulation). fNIRS monitored HbO_2_ concentration changes in bilateral prefrontal and parietal cortices to identify significantly activated brain regions (e.g., left inferior frontal gyrus, bilateral precentral gyrus). ② Treatment guidance (non-real-time feedback): Rehabilitation protocols were adjusted based on fNIRS results from D35 (e.g., increasing the frequency and duration of sensory electrical stimulation). During subsequent treatments, fNIRS was not used as real-time feedback. A follow-up fNIRS scan was conducted after D77 rehabilitation to verify improvements in cortical activation. To evaluate cortical activation under different sensory modalities, the fNIRS task in this study included five types of oropharyngeal stimuli: neuromuscular electrical stimulation, small brush stimulation, sensory electrical stimulation, pneumatic pulse stimulation, and oral vibrator stimulation.

Each task was repeated five times. A baseline period of 0–2 s was used to compute the mean HbO_2_ concentration change for each task interval, which was then averaged and compared across task types to assess differences in cortical activation.

Prior to the assessment, the patient was positioned comfortably and rested for 10 min. Detailed instructions were provided, and a quiet, distraction-free environment was ensured. Throughout the procedure, the patient wore a properly positioned fNIRS cap and completed tasks as guided by a computer interface (Figure 3).

Each task was set to last 20 s, followed by a 30 s resting interval, repeated five times, for a total session duration of 250 s. After each task set, the headgear was removed, and a 10 min rest was provided to minimize cross-task interference and ensure data independence.

Continuous recording of cerebral hemodynamic responses during swallowing tasks showed that peak oxygenated hemoglobin (HbO_2_) concentrations in the inferior frontal gyrus and precentral gyrus increased significantly during sensory electrical stimulation tasks (Figure 4). This indicated robust activation of relevant cortical areas, whereas other tasks elicited relatively weaker responses. These findings were subsequently used to adjust the therapeutic plan accordingly (Supplementary Table S5).

## Outcomes

### Swallowing function recovery

The patient's swallowing ability demonstrated significant multidimensional improvement following the optimization of the rehabilitation protocol guided by fNIRS. The Functional Oral Intake Scale (FOIS) score improved from level I (total dependence on tube feeding) at admission to level VI on day 77 (able to consume soft textures orally), indicating the restoration of independent oral intake. The Penetration-Aspiration Scale (PAS) score improved markedly from 8 (severe aspiration) to 2 (mild penetration), reflecting a substantial enhancement in swallowing safety. The Modified Barium Swallow Impairment Profile (MBSImP) total score decreased from 49 to 28 (Supplementary Table S6), with the most notable improvements observed in oral preparation, soft palate control, and pharyngeal constriction components.

### Video fluoroscopic analysis of swallowing dynamics

Swallowing kinematic parameters, as observed in the video fluoroscopic images, revealed substantial functional recovery:

Pharyngeal constriction ratio increased from 34% to 74%, representing an approximate 117% improvement.
Pharyngeal transit time was reduced from 120 s to 3 s (a 97.5% decrease).
Hyoid bone displacement increased from 110% to 136% of the C2–C4 distance.
These findings suggest significant enhancement in oropharyngeal coordination and overall swallowing biomechanics.

### Nutritional status improvement

The patient's body mass index (BMI) increased from 16.15 kg/m^2^ at baseline to 17.03 kg/m^2^, indicating a notable trend toward nutritional recovery and improved caloric intake.

### Improvement in saliva control

Substantial improvements in saliva management were observed:

Subjective drooling severity decreased from “severe” (score of 4) to “mild” (score of 1).
Unstimulated salivary flow rate decreased from 0.45 g/min to 0.18 g/min.
Murray secretion severity scale improved from grade 3 to grade 1 on fiberoptic endoscopic examination.
These findings reflect enhanced oral motor control and salivary regulation.
Weaning from Tubes and Functional Restoration: Based on the comprehensive improvements in swallowing function, safety, and treatment tolerance, the patient was successfully weaned from both the nasogastric tube and tracheostomy cannula on day 77. Independent respiration and oral intake were fully restored. The Water Swallow Test improved from level V to level II, with no signs of overt aspiration or coughing during water or soft food consumption. This indicates near-complete recovery of oral and pharyngeal phase coordination (Supplementary Table S7).

## Discussion

Dysphagia is a frequent complication in patients with severe traumatic brain injury (sTBI), with an incidence ranging from 38% to 65% (9). When injury involves the medullary swallowing centers—such as the nucleus tractus solitarius or nucleus ambiguous-or disrupts corticobulbar pathways, patients often present with hallmark symptoms including loss of the pharyngeal reflex, delayed swallow initiation, inadequate hyolaryngeal excursion, and silent aspiration (10). Although a variety of conventional rehabilitation techniques (e.g., thermal-tactile stimulation, surface electromyography biofeedback) are commonly employed, two major limitations persist: (1) substantial variability in treatment efficacy across individuals, and (2) the lack of real-time feedback on cortical neurofunction, resulting in 45%–60% of patients failing to achieve functional swallowing recovery (11).

Functional near-infrared spectroscopy (fNIRS) offers a non-invasive method to monitor hemodynamic changes in cortical regions by detecting oxygenated hemoglobin (HbO_2_) concentration shifts. It has emerged as a valuable neuroimaging tool for assessing cortical involvement in swallowing-related tasks and has the potential to guide individualized neurorehabilitation planning (12). Recent studies support the feasibility of using fNIRS to evaluate brain activation patterns during specific swallowing tasks and to estimate cortical responsiveness and recovery potential (13).

In the present case, we report a 15-year-old male with persistent neurogenic dysphagia following sTBI. Despite undergoing a comprehensive 35-day standardized rehabilitation program incorporating multimodal interventions, the patient showed minimal functional improvement in swallowing, as indicated by an MBSImP total score improvement of less than 5% and persistent PAS score of 8. This raised concerns about insufficient cortical activation or limited task specificity in the intervention.

fNIRS-based assessment revealed significant increases in HbO_2_ concentration in the left inferior frontal gyrus (Broca's area) and bilateral precentral gyri (primary motor cortex) during sensory electrical stimulation, suggesting that this task elicited meaningful cortical engagement. Based on these neurophysiological insights, the rehabilitation protocol was adapted to emphasize stimulation modalities that activated target brain regions, while eliminating ineffective or non-responsive components.

Following 42 days of fNIRS-guided therapy, the patient demonstrated marked improvements in swallowing function. FOIS scores improved from level I (tube-dependent) to level VI (oral intake of soft solids), and PAS scores decreased from 8 (severe aspiration) to 2. By day 77, the patient was successfully weaned from both the nasogastric feeding tube and tracheostomy, regaining independent oral intake and spontaneous breathing.

This case underscores the clinical utility of fNIRS as a real-time neurofunctional feedback tool for guiding personalized rehabilitation in patients with refractory neurogenic dysphagia. Unlike conventional “trial-and-error” approaches, fNIRS enables objective assessment of task-related cortical activation, enhancing therapeutic precision and potentially improving clinical outcomes.

Nevertheless, several limitations warrant consideration. First, the lack of long-term follow-up in this case precludes conclusions about the durability of treatment effects. Second, fNIRS is inherently limited in its ability to assess deep brain structures such as the brainstem, which play a critical role in swallowing control. Lastly, this was a single-case observation; larger cohort studies and multicenter trials are needed to validate the clinical applicability, reproducibility, and generalizability of fNIRS in neurogenic dysphagia rehabilitation.

## Conclusion

This case highlights the complexity of managing severe post-TBI dysphagia, particularly in patients unresponsive to conventional multimodal rehabilitation and pharmacological interventions. Functional near-infrared spectroscopy (fNIRS) provided objective cortical activation mapping, enabling precision adjustments to the therapeutic plan and facilitating clinically meaningful recovery within a relatively short rehabilitation window. Beyond its role as a neuroimaging tool, fNIRS demonstrated potential as a bedside neurofeedback modality to guide individualized intervention strategies.

Given the heterogeneity of post-TBI dysphagia and its profound impact on nutrition, airway safety, and overall recovery, integrating neurophysiological monitoring into routine assessment may improve patient stratification, optimize treatment allocation, and enhance outcomes. Longitudinal follow-up with standardized swallowing assessments and neuroimaging is essential to evaluate the durability of recovery, monitor for relapse, and further refine precision rehabilitation protocols.

## Data Availability

The original contributions presented in the study are included in the article/Supplementary Material, further inquiries can be directed to the corresponding author.

## References

[1] Capizzi A Woo J Verduzco-Gutierrez M. Traumatic brain injury: an overview of epidemiology, pathophysiology, and medical management. Med Clin North Am. (2020) 104:213–38. 10.1016/j.mcna.2019.11.001 32035565

[2] Khellaf A Khan DZ Helmy A. Recent advances in traumatic brain injury. J Neurol. (2019) 266:2878–89. 10.1007/s00415-019-09541-4 31563989 PMC6803592

[3] Morgan A Ward E Murdoch B. Clinical progression and outcome of dysphagia following paediatric traumatic brain injury: a prospective study. Brain Inj. (2004) 18:359–76. 10.1080/02699050310001617424 14742150

[4] Lee WK Yeom J Lee WH Seo HG Oh BM Han TR. Characteristics of dysphagia in severe traumatic brain injury patients: a comparison with stroke patients. Ann Rehabil Med. (2016) 40:432–9. 10.5535/arm.2016.40.3.432 27446779 PMC4951361

[5] Won SY Krieger S Dubinski D Gessler F Behmanesh B Freiman T Neurogenic dysphagia in subdural hematoma. Front Neurol. (2022) 12:701378. 10.3389/fneur.2021.701378 35153966 PMC8826688

[6] Halfpenny R Stewart A Kelly P Conway E Smith C. Dysphagia rehabilitation following acquired brain injury, including cerebral palsy, across the lifespan: a scoping review protocol. Syst Rev. (2021) 10:312. 10.1186/s13643-021-01861-9 34903269 PMC8667523

[7] Howle AA Baguley IJ Brown L. Management of dysphagia following traumatic brain injury. Curr Phys Med Rehabil Rep. (2014) 2:219–30. 10.1007/s40141-014-0064-z

[8] Warnecke T Labeit B Schroeder J Reckels A Ahring S Lapa S Neurogenic dysphagia: systematic review and proposal of a classification system. Neurology. (2021) 96:e876–89. 10.1212/WNL.0000000000011350 33318164

[9] Wen X Peng J Zhu Y Bao X Wan Z Hu R Hemodynamic signal changes and functional connectivity in acute stroke patients with dysphagia during volitional swallowing: a pilot study. Med Phys. (2023) 50:5166–75. 10.1002/mp.16535 37314082

[10] Bérubé M Ouellet S Turcotte V Gagné A Gélinas C. Adaptation and validation of the standardized swallowing assessment tool for patients with moderate-severe traumatic brain injury and cervical spinal cord injury. J Neurotrauma. (2024) 41:1101–11. 10.1089/neu.2022.0418 37725567

[11] Ayaz H Baker WB Blaney G Boas DA Bortfeld H Brady K Optical imaging and spectroscopy for the study of the human brain: status report. Neurophotonics. (2022) 9(S2):S24001. 10.1117/1.NPh.9.S2.S24001 36052058 PMC9424749

[12] Chua DMN Chan KM. Cortical activation during swallowing exercise tasks: an fNIRS pilot study. Dysphagia. (2025) 40:327–35. 10.1007/s00455-024-10730-1 38980390 PMC11893666

[13] Knollhoff SM Hancock AS Barrett TS Gillam RB. Cortical activation of swallowing using fNIRS: a proof of concept study with healthy adults. Dysphagia. (2022) 37:1501–10. 10.1007/s00455-021-10403-3 35132474

