# Supplementary material for: Case Report: fNIRS-guided rehabilitation in refractory post-traumatic dysphagia
Source: Front Rehabil Sci. 2025 Nov 26;6:1712962. doi: 10.3389/fresc.2025.1712962 (PMC12689878; doi:10.3389/fresc.2025.1712962)
Supplement: Supplementary file 1 [file Table1.docx]

**Table 1 Frenchay Dysarthria Assessment**

| Evaluation time | FDA-2 sub-items (all E-level = severely impaired, D-level = moderately impaired, C-level = mildly impaired) | Uncontrollable jaw movement / teeth grinding |
| --- | --- | --- |
| D0（Admission） | Oral and facial structure (E), respiratory support (E), laryngeal function (E), related reflexes (E), articulation (E), resonance (E) | Persistent with activity frequency ≥5 times per minute, accompanied by noticeable bruxism sounds |
| D35（End of first stage） | Oral and facial structure (E), respiratory support (E), laryngeal function (D), related reflexes (D), articulation (E), resonance (E) | The activity frequency drops to 3-4 times per minute, and the grinding sound weakens |
| D49（Mid-phase of stage 2） | Oral and facial structure (D), respiratory support (D), laryngeal function (C), related reflexes (C), articulation (D), resonance (D) | The activity frequency drops to 1-2 times per minute, and no obvious grinding sound is heard |
| D77（Recovery complete） | Oral and facial structure (C), respiratory support (C), laryngeal function (C), related reflexes (C), articulation (C), resonance (C) | Occasional (<1 times per minute), disappears when quiet |
