# Supplementary material for: Case Report: fNIRS-guided rehabilitation in refractory post-traumatic dysphagia
Source: Front Rehabil Sci. 2025 Nov 26;6:1712962. doi: 10.3389/fresc.2025.1712962 (PMC12689878; doi:10.3389/fresc.2025.1712962)
Supplement: Supplementary file 2 [file Table2.docx]

**Table 2 Drooling Assessment**

| Parameter | Baseline | 35day | 49day | 77 day |
| --- | --- | --- | --- | --- |
| Subjective Drooling Score | 4 | 4 | 3 | 1 |
| Saliva Flow Rate | 0.45 | 0.43 | 0.32 | 0.18 |
