# Supplementary material for: Case Report: fNIRS-guided rehabilitation in refractory post-traumatic dysphagia
Source: Front Rehabil Sci. 2025 Nov 26;6:1712962. doi: 10.3389/fresc.2025.1712962 (PMC12689878; doi:10.3389/fresc.2025.1712962)
Supplement: Supplementary file 3 [file Table3.docx]

**Table 3 Video fluoroscopic Swallowing Analysis Results**

| Swallowing Parameter | Baseline | 35day | 49day | 77 day |
| --- | --- | --- | --- | --- |
| Oral Transit Time(s) | ND | ND | 60 | 13 |
| Pharyngeal Transit Time(s) | 120 | 112 | 21 | 3 |
| Hyoid displacement(%) | 110 | 112 | 125 | 136 |
| Pharyngeal Constriction Ratio(%) | 34 | 32 | 69 | 74 |
| PAS | 8 | 8 | 5 | 2 |
