# Supplementary material for: Case Report: fNIRS-guided rehabilitation in refractory post-traumatic dysphagia
Source: Front Rehabil Sci. 2025 Nov 26;6:1712962. doi: 10.3389/fresc.2025.1712962 (PMC12689878; doi:10.3389/fresc.2025.1712962)
Supplement: Supplementary file 4 [file Table4.docx]

**Table 4 Fiberoptic Endoscopic Evaluation of Swallowing**

| Parameter | Baseline | 35day | 49day | 77 day |
| --- | --- | --- | --- | --- |
| Murray Secretion Scale | 3 | 3 | 2 | 1 |
| FEDSS | 6 | 6 | 4 | 1 |
