# Supplementary material for: Case Report: fNIRS-guided rehabilitation in refractory post-traumatic dysphagia
Source: Front Rehabil Sci. 2025 Nov 26;6:1712962. doi: 10.3389/fresc.2025.1712962 (PMC12689878; doi:10.3389/fresc.2025.1712962)
Supplement: Supplementary file 5 [file Table5.docx]

**Table 5 Rehabilitation Outcomes After Precision Adjustment of Treatment Plan Based on fNIRS Evaluation**

| **Treatment stage** | **Model** | **Sessions per week** | **Total sessions** | **Avg. session duration (min)** | **Cumulative therapy duration (min)** |
| --- | --- | --- | --- | --- | --- |
| **D0-D35**  **Pre-treatment** | Sensory Electrical Stimulation (SES) | 5 | 35 | 10 | 350 |
|  | Catheter Balloon Dilation and Pharyngeal Pressure Biofeedback Training | 5 | 35 | 5 | 175 |
|  | Air-Pulse Stimulation | 5 | 35 | 10 | 350 |
|  | Oral Vibratory Stimulation | 5 | 35 | 10 | 350 |
|  | Low-Frequency Electrical Stimulation of the Masseter Muscle | 5 | 35 | 20 | 700 |
|  | Tongue Suction Training | 5 | 35 | 5 | 175 |
|  | Targeted Low-Frequency Stimulation of Suprahyoid Muscles | 5 | 35 | 20 | 700 |
|  | Beckman Oral Motor Intervention (BOMI) | 5 | 35 | 10 | 350 |
|  | Oral Sensory Localization Stimulation | 5 | 35 | 10 | 350 |
| **D36-D77Post-treatment** | Sensory Electrical Stimulation (SES) | 5 | 40 | 10 | 400 |
|  | Oral Sensory Localization Stimulation | 5 | 40 | 5 | 200 |
|  | Air-Pulse Stimulation | 5 | 40 | 10 | 400 |
|  | Targeted Low-Frequency Stimulation of Suprahyoid Muscles | 5 | 40 | 10 | 400 |
|  | Beckman Oral Motor Intervention (BOMI) | 5 | 40 | 10 | 400 |
|  | Catheter Balloon Dilation and Pharyngeal Pressure Biofeedback Training | 5 | 40 | 10 | 400 |
