# Supplementary material for: Case Report: fNIRS-guided rehabilitation in refractory post-traumatic dysphagia
Source: Front Rehabil Sci. 2025 Nov 26;6:1712962. doi: 10.3389/fresc.2025.1712962 (PMC12689878; doi:10.3389/fresc.2025.1712962)
Supplement: Supplementary file 6 [file Table6.docx]

**Table 6 Longitudinal MBSImP Score Summary**

| Parameter | Baseline | 35day | 49day | 77 day |
| --- | --- | --- | --- | --- |
| Lip Closure | 4 | 4 | 4 | 2 |
| Tongue Control | 3 | 3 | 2 | 1 |
| Bolus Preparation | 3 | 3 | 2 | 2 |
| Bolus Transport | 4 | 4 | 3 | 3 |
| Oral Residue | 4 | 4 | 3 | 2 |
| Initiation of Pharyngeal Swallow | 4 | 4 | 3 | 3 |
| Soft Palate Elevation | 4 | 4 | 3 | 3 |
| Laryngeal Elevation | 3 | 3 | 2 | 2 |
| Hyoid Excursion | 2 | 2 | 1 | 1 |
| Epiglottic Movement | 2 | 2 | 2 | 1 |
| Laryngeal Vestibule Closure | 2 | 2 | 1 | 1 |
| Pharyngeal Stripping Wave | 2 | 2 | 1 | 1 |
| Pharyngeal Contraction | 3 | 3 | 1 | 1 |
| PES Opening | 2 | 2 | 1 | 1 |
| Tongue Base Retraction | 4 | 3 | 3 | 2 |
| Pharyngeal Residue | 3 | 2 | 2 | 2 |
| Esophageal Clearance in Upright Position | 0 | 0 | 0 | 0 |
| Total Score | 49 | 47 | 35 | 28 |
