# Supplementary material for: Case Report: fNIRS-guided rehabilitation in refractory post-traumatic dysphagia
Source: Front Rehabil Sci. 2025 Nov 26;6:1712962. doi: 10.3389/fresc.2025.1712962 (PMC12689878; doi:10.3389/fresc.2025.1712962)
Supplement: Supplementary file 7 [file Table7.docx]

**Table 7 Follow-up after 3 and 6 months**

| Follow-up time | FOIS | PAS | BMI (kg/m²) | Adverse Event |
| --- | --- | --- | --- | --- |
| Three months  (D167) | VI (can eat soft food normally without assistance) | Level 2 (light infiltration, no cough) | 17.52±0.3 | No aspiration pneumonia, aspiration and other events |
| 6 months (D257) | VI (You can eat soft food and swallow normally) | Grade 2 (no infiltration aggravation) | 17.85±0.2 | No regressive swallowing, no other complications |
